# Cross sectional survey of maternal and newborn quality of care in Niger: Selected findings, lessons learned and recommendations

**DOI:** 10.1371/journal.pgph.0003268

**Published:** 2024-11-13

**Authors:** Alexandre Quach, Barbara Madaj, Katie Fahy, Aminata Tinni Konate, Ibrahim Souley, Lucien Omar Marcel, Adama Traore, Monir Islam, Uzochukwu Egere, Charles Anawo Ameh

**Affiliations:** 1 Department of International Public Health, Liverpool School of Tropical Medicine, Liverpool, United Kingdom; 2 Department of Public Health, Policy and Systems, University of Liverpool, Liverpool, United Kingdom; 3 Ministère de la Santé Publique, de la Population et des Affaires Sociales, Niamey, Niger; 4 Department of Obstetrics and Gynaecology, University of Nairobi, Nairobi, Kenya; PLOS: Public Library of Science, UNITED STATES OF AMERICA

## Abstract

Comprehensive assessments of quality of care (QoC) are essential for monitoring progress towards meeting global and national maternal and newborn health (MNH) targets. Liverpool School of Tropical Medicine (LSTM) and the Niger Ministry of Public Health adapted a 2014 WHO QoC tool to cover antenatal care (ANC) and postnatal care (PNC) and include client voices through exit interviews. The objectives of the study were to assess quality of MNH care in Niger and to document lessons learnt from implementing the LSTM QoC tool. Quality of Care (QoC) was assessed using five tools: health facility survey (using register and official record data), partograph reviews, healthcare provider knowledge assessment, exit interviews with clients, and observation of antenatal and postnatal care. A nationally representative sample of 110 public healthcare facilities at three levels (integrated health centres, district hospitals and mother-child hospitals) and 2153 women were included. Descriptive analysis with 95% confidence intervals was presented. The health facility survey showed variable access to electricity (63% [53–72]), water (72% [62–81]), and transportation (40% [31–50]). Tests and treatment for HIV, TB, and malaria were found in more than 90% of relevant facilities. During observation of first ANC visit, 62% [48–74] women were tested for HIV; 13% [5–30] for pre-eclampsia and 22% [12–36] for anaemia. Observation of PNC showed much lower rates of screening (15% [7–27] tested for HIV and 4% [0;11] for malaria). Partographs were used in 86% of deliveries with inconsistent completion. At client interviews, respectful care indicators were variable, with providers not always explaining results to clients (59% [50–67]). Targeted allocation of resources and training could impact on QoC and reduce missed opportunities for prevention, screening and management of diseases among pregnant women and babies. The QoC assessment tool proved capable of offering a comprehensive overview of priorities in MNH, while maintaining feasibility in the Nigerien context.

## Background

High-quality maternal, newborn health (MNH) is essential for the achievement of health-related Sustainable Development Goal 3 (SDG3) and Universal Health Coverage (UHC) [1]. Globally, there has been a marked shift from focusing on coverage alone to ‘effective coverage’, which aims to put ‘content into contact’, and is defined by Marsh et al’s (2020) as ‘the proportion of a population in need of a service that resulted in a positive health outcome from the service’ [2]. Achieving positive health outcomes requires improving availability as well as quality of care offered [3, 4], which faces challenges in several countries across sub-Saharan Africa, including Niger. Despite the considerable progress in Niger’s MNH targets, such as achieving the Millennium Development Goal 4 (MDG4) target of reducing child mortality by two thirds in 2015, there is still ample room for improvement. The latest Demographic Health Survey (DHS) showed that the maternal mortality ratio was 535 per 100,000 live births between 2005 and 2012 and the newborn mortality rate was 24 per 1,000 live births between 2008 and 2012 [5]. Less than 33% of women attended 4 antenatal care (ANC) visits, while 85% of neonates had no postnatal care (PNC) visit [5]. Based on the 2012 DHS report, 30% of women gave birth in healthcare facilities [5]. When attending health facilities during and after pregnancy, women may not receive all the recommended services, including the integrated screening and management for infectious diseases such as HIV, tuberculosis (TB) and malaria, which was reiterated in the 2011–2015 Nigerien Health Development Plan [6].

Against this backdrop, the Nigerien Ministry of Public Health (Ministère de la Santé Publique–MSP) requested a detailed assessment of the quality of care (QoC) in Niger towards the formulation of a targeted national strategy for reducing maternal and child mortality and morbidity. In 2016 a partnership was created with the Liverpool School of Tropical Medicine (LSTM) who provided technical assistance, funded by Save the Children International, with financing from the Global Fund to fight AIDS, tuberculosis and malaria, to support this endeavour.

At the time of the study in Niger, health care was delivered through three national hospitals, one national maternity hospital (MIG—Maternité Issakia Gobi), six regional hospitals (CHR—Centres Hospitaliers Régionaux), seven mother-child hospitals (CSME—Centres de Santé Mère Enfant), and 33 district hospitals (HD–Hôpitaux de district) that are expected to deliver Comprehensive Emergency Obstetric and Newborn Care (CEmONC). At the peripheral level, health care was provided by 241 Integrated Health Centres (IHC) (CSI–Centres de Santé Intégrés) type II, 648 IHC type I and 2,523 health posts. Integrated Health Centres type II are in urban areas and include a maternity unit, while Integrated Health Centres type I are located in rural areas and do not necessarily include a maternity unit. Both are expected to deliver Basic Emergency Obstetric and Newborn Care (BEmONC) [7]. In addition to this, the private sector consists of 299 healthcare facilities, including six private hospitals, 45 clinics and 248 medical practices [7].

Based on the healthcare organisation, non-complicated cases are managed at primary healthcare level (IHC), while those clients who require more specialist support are referred to and looked after at higher-level facilities (DH and above). Standard ANC and PNC clinics are not offered at those dealing with complicated cases only, but clients enrolled in more specialist care may be offered ANC and PNC clinics in the higher-level facilities [8]. Focused antenatal care (FANC) approach emphasises the quality of care given during each visit instead of focusing on the number of visits. As a result, although WHO recommended eight ANC contacts, at time of survey the Nigerien MSP scheduled four visits as follows: by the end of 16 weeks of pregnancy, then between 24 and 28 weeks of pregnancy, at 32 weeks, and finally at 36 weeks [9]. Women who have complications or conditions that require specialist treatment may require additional visits. Postnatal services were offered as part of intrapartum care for those giving birth in facilities for the immediate postnatal period (in-patient) and in outpatient clinics for all other clients [10]. In Niger, practice described by the MSP at the time of the study for PNC covered care for up to 42 days post giving birth, at two standard timepoints: early visits on day 6 and late visit on day 42.

The objective of this study was to assess QoC in MNH in a nationally representative sample of healthcare facilities in Niger. While the assessment generated in-depth insight into services and care, this manuscript focuses on the analysis of selected aspects only. In addition to the findings, insights into the usability and recommendations from using the QoC assessment tool co-created with the Nigerien Ministry of Health are also presented here.

## Methods

This was a cross-sectional study assessing the quality of care offered as part of the antenatal, intrapartum, postnatal and paediatric care services in Niger.

### Sample

To ensure representation across levels of care, facilities from peripheral, regional and central levels were included. However, not all facilities in the structures provided maternity services and therefore the assessment excluded those from the sampling [7]. A hundred and ten (110) public healthcare facilities with at least 20 births per quarter were randomly selected from the national database of healthcare facilities. This minimum threshold was applied to allow for the service use to be of high enough volume to assess quality of care provided. Sampling was stratified by facility level and regions, comprising all eight administrative regions in Niger (Agadez, Diffa, Dosso, Maradi, Niamey, Tahoua, Tillabéri and Zinder). The stratified sample comprised randomly selected 79 out of 430 integrated health centres (IHC) and 22 out of 33 district hospitals (DH) at the peripheral level, and all nine higher-level healthcare facilities (Mother-Child hospitals -MCH- plus the national maternity hospital) from the regional and central levels. With this sample size national estimates would not exceed a 10% margin of error.

At the time of the study, partners at the Ministry of Public Health described ANC and PNC services in Niger as mainly provided in IHC facilities at the peripheral level, with a few exceptions in certain HDs and CSMEs. Less than 10% of HD and half of upper-level facility provided ANC. Thus, data from HD and CSME are of limited representativity with no rooms or services to examine and no estimates could be generated from our results, especially for ANC. However, because even without a designated ward or officially attributed service, some women would still receive ANC or PNC in certain CEmONC facilities (e.g. women followed for high-risk pregnancies, postnatal care for complicated deliveries etc.) and interviews were conducted whenever these women were found in a healthcare facility. The same would apply for observations (planned in 31 selected CSIs and all HD and upper-level facilities).

### Tools and procedure

The 2015 WHO Quality of Care Framework for maternal and newborn health comprises health system as basis for how care is delivered (provision) and received (experience), leading to outcomes [11, 12]. These elements are aligned to Donabedian structure, process and outcomes (SPO) measures providing a useful set up for reframing facility assessments [13]. However, many of the international tools used (WHO Service Availability and Readiness Assessment (SARA), the World Bank Service Delivery Indicators (SDI) and the Service Provision Assessment (SPA) for the DHS) at time of the survey focused predominantly on availability and readiness to provide care (structure). Of note, these tools have since been updated to incorporate a more considerable QoC focus compared to the time of the study [14, 15]. Thus, the tool used for this study was developed by adapting a 2014 WHO QoC tool [16] and incorporating elements of other tools and indicators [17–25].

It was co-developed with the Directorate for Mother and Child Health (Direction de la Santé Mère Enfant—DSME) at MSP. Usability and relevance of the tool was ensured by integrating existent national guidelines on standards of care or when not available through the conduct of MNH stakeholder workshop to identify appropriate variables [8].

The tool assessed the full continuum of care utilising a range of SPO indicators and comprised five sub-tools covering the availability and experience of care: a healthcare facility survey (HFS), partograph reviews, knowledge assessment for healthcare providers, client exit interviews of antenatal, intrapartum, postnatal and paediatric care; and direct observation of provision of antenatal and postnatal care. While not directly focusing on family planning (FP), these services were assessed as part of antenatal and postnatal care. Although the health facility survey covered both in and outpatient services, the experience of care assessment focused on outpatient clients only, except for intrapartum service exit interviews.

The HFS was completed in all facilities and included modules corresponding to specific, relevant, areas: A) general characteristics, B) antenatal care, C) intrapartum care, and D) postnatal care, E) paediatric inpatient care, and F) paediatric outpatient care. Data collectors assessed the presence (availability) of basic infrastructure, essential medicines, equipment and supplies, and retrieved data on the uptake of services from registers and relevant official records. The data for one, most recently completed, month–March 2018 –was used in the assessment to allow for the detailed records to be reviewed during the visit.

For all other components, sampling was used to determine the required number of respondents to estimate the prevalence of the characteristics with a specified margin of error allowance. Specifically, in each facility, up to ten partographs filled in the reference month were reviewed. In the event where more than ten were available, these were randomly selected. Knowledge assessment in the form of multiple-choice questionnaires was used for up to four healthcare providers per facility, with respondents selected purposefully to include as wide as possible range of providers working across services and representing different cadres. Client exit interviews, covering process and respectful maternity care indicators to assess experience of care were conducted with women receiving care for themselves or their child at antenatal, intrapartum, postnatal and paediatric care (five women per type of visit, totalling 20 in each facility).

Due to resource restrictions, direct observation was limited to antenatal and postnatal care services in a stratified random sample of 31 integrated health centres, 22 districts hospitals and nine higher-level facilities, totalling 62 healthcare facilities (see S1 Table). Within each selected facility, seven observations each of ANC and PNC were conducted.

All clients and healthcare providers meeting the eligibility criteria within the selected services and present at the time of the facility assessment were eligible for recruitment. As pregnant women and mothers seeking care in Niger may be under 18 and attend alone, it was considered ethically acceptable for them to provide their own consent (emancipated minors). This was essential to capture the quality of care provided to all clients.

All respondents went through an informed consent process prior to data collection. For client assessments (exit interviews and observation) in particular, due to low literacy rate (at 19% for women in Niger involvement of independent witnesses was required for those who needed support with reading and signing the consent). The pool of potential witnesses did not exclude healthcare providers working at the facility. With the assessment being independent of service provision, potential pressure for clients to agree to take part because of a member of facility staff being a witness was deemed minimal. Also, the availability of literate people to witness consenting was limited, at times making healthcare providers the only source of help in the process. Based on exit interview respondents, four in five consent forms involved a witness (79%). In addition to knowledge assessment, healthcare providers went through the consenting process for observation, but the process of seeking consent was done separately to client consenting for that activity. For register and record reviews, signed consent was sought from heads of facilities.

The final tool was reviewed with Nigerien stakeholders and pilot-tested in two healthcare facilities in Niamey at the end of March 2018. Recruitment and data collection took place between May 3^rd^ and June 13^th^ 2018, by 40 data collectors with medical or social science background (only medically trained data collectors were involved in observation) in teams of four. Any reviewed document including identifiable data, such as partographs, was treated as confidential, and fully anonymized during data collection. Data were collected using an electronic platform on ‘Survey CTO’ on Android tablets, with inbuilt validations and filters for improved quality [26]. The quality assurance process, including extensive checks with healthcare facilities to clarify any queries, minimised the risk of measurement error. On completion, forms were submitted to the LSTM server for processing. All forms were quality checked and where queries were raised, these were addressed in communication with teams on the ground before finalising datasets.

### Analysis

Service availability and readiness were analysed in four domains: provision of care, basic infrastructure, integrated services, and essential medicines. The analysis was performed using R statistical software version 3.5.1 [27]. Descriptive results were stratified by type of facility (IHC, DH and MCH). Ninety-five percent confidence intervals (95% CI) are indicated in brackets after every result. Where the facilities were too few to derive a CI 95%, an asterisk (*) is used following the proportion result.

A dashboard was constructed to offer a visual representation of the key results covered as part of the assessment. Although not the focus of this paper, the dashboard was stratified by regions to help as decision-making tool for national policy makers. The dashboard covered structure and process indicators representing the core areas of focus in assessing quality of care. Indicators linked to staffing levels defined sufficient numbers of specific healthcare professionals according to the “Norms and Standards for Infrastructure, Equipment and Staff of the National Health System in Niger” document from October 2006, not available online [8]. For each region and type of facility, the proportion of healthcare facilities providing each key-service was summarized in one of four categories: green (76% to 100%); yellow (51% to 75%); amber/orange (26% to 50%); and red (0% to 25%).

### Ethical approval

This study received approval from the National Ethics Committee for Health Research in Niger (Comité National d’Ethique pour la recherche en santé—CNERS) and the institutional research ethics committee at LSTM (LSTM Research Ethics Committee—LSTM REC).

## Results

All selected healthcare facilities (79 IHCs, 22 DHs and 9 MCHs) were surveyed. A total of 968 partographs were reviewed, 369 knowledge tests completed, 1679 clients interviewed at different section of the continuum of maternal, newborn and child care; and 474 consultations (250 in ANC and 224 in PNC) observed. The bulk of client data came from lower-level facilities. For exit interviews that was at 92% for ANC, 63% for Intrapartum, 89% for PNC and 74% for paediatric care. Similarly, 95% ANC and 79% PNC observation clients attended IHC. An overview of the type of data collected by level of care is presented in S2 Table.

### Availability of services

Regarding basic infrastructure, 63% [53–72] of healthcare facilities always had electricity and 72% [62–81] had access to clean water without interruption. For referral systems, 40% [31–50] had means of transporting patients available in the facility (Table 1). ANC and PNC were both offered in all IHCs. Only some DHs and MCHs provided ANC and PNC services, usually for complicated cases: ANC was provided in 9% [3–25] of DHs and 44%* of MCHs, while PNC was provided in 31% [14–54] of DH and 67%* of MCH (Table 1).

**Table 1:** Percentage (95% CI) of facilities with Infrastructure available and services routinely provided, by healthcare facility type.

	IHC (N = 79)	DH (N = 22)	MCH (N = 9)	All (N = 110)
**Infrastructure**				** **
Electricity always available	61 [50–71]	89 [63–98]	89*	63 [53–72]
Clean water always available	71 [59–80]	86 [63–96]	100*	72 [62–81]
Transportation for referral available at facility	35 [25–45]	97 [78–100]	89*	40 [31–50]
**Complementary examination facilities**				
Laboratory available	19 [11–29]	100 [100–100]	100*	26 [19–35]
Radiography available	0 [0–0]	38 [19–62]	67*	4 [2–7]
Echography available	0 [0–0]	72 [49–87]	100*	7 [5–10]
**Maternal, newborn and child care services**				
Provision of ANC	100 [100–100]	9 [3–25]	44*	93 [89–95]
Provision of PNC	100 [100–100]	31 [14–54]	67*	95 [91–97]
Performance of C-sections	0 [0–0]	83 [59–94]	100*	8 [5–11]
Neonatal unit/space	0 [0–0]	89 [63–98]	100*	92 [72–98]
Paediatric care (outpatient)	100 [100–100]	86 [64–96]	89*	99 [97–100]
Paediatric care (inpatient)	0 [0–0]	100 [100–100]	89*	98 [97–98]

*No CI provided as statistics are without error and reflect true value (all existent MCH nationwide are included in the sample)

N = number of facilities

### Availability of testing kits, drugs and vaccines

Availability of consumables for integrated services in ANC and PNC varied across facilities and are displayed in Table 2. Means of testing for HIV and malaria were available in most facilities (92% [84–96] and 96% [92–98], respectively). Sputum tests for TB were available in all DHs, reflecting the national policy. Other means for screening were usually available at higher level facilities either by rapid testing or routine laboratory, but less available at IHC level: haemoglobin tests were found in 13% [7–22] and syphilis rapid test in 13% [8–23] of IHCs. There was variable availability of essential medicines at all types of facilities. Sulfadoxine-pyrimethamine for Intermittent Preventive Treatment during pregnancy (IPTp) and Artemisinin-based combination treatment (ACT) were found in most facilities (91% [83–96] and 70% [60–79], respectively). Quinine was available in 9 out of 10 facilities (90 [82–95]). First-line antiretroviral (ARV) combination for HIV and anti-TB treatment were concentrated in DHs (91% [66–98] and 94% [76–99], respectively), while being less consistently found at other types of facilities (Table 2).

**Table 2:** Percentage (95% CI) of facilities with tests and medicines for screening, diagnosis and management of diseases available, by healthcare facility type.

	IHC (N = 79)	DH (N = 22)	MCH (N = 9)	All (N = 110)
Diagnostic tests				
HIV rapid test	91 [82–96]	100 [100–100]	78*	92 [84–96]
HIV lab test	7 [3–16]	79 [52–93]	100*	14 [9–21]
Rapid diagnostic test for syphilis	13 [8–23]	92 [70–98]	100*	21 [14–29]
Sputum tests for TB	34 [25–46]	100 [100–100]	22*	39 [29–49]
Rapid diagnostic test for malaria	99 [91–100]	72 [47–88]	78*	96 [92–98]
Thick blood smear	10 [5–18]	97 [78–100]	100*	17 [12–25]
Haemoglobin test	13 [7–22]	79 [55–92]	100*	19 [13–27]
Urine analysis (dip stick)	54 [43–65]	100 [100–100]	89*	58 [48–68]
**Medicines for prevention and management of infectious diseases**				
ARV first line combination for HIV	28 [19–38]	91 [66–98]	89*	33 [25–43]
HRZE quadritherapy for TB	36 [26–46]	94 [76–99]	0*	39 [30–49]
Sulfadoxine pyrimethamine	91 [83–96]	67**	100*	91 [83–96]
ACT or artesunate/arthemeter IM	91 [83–96]	89 [68–97]	100*	91 [84–96]
Quinine	91 [82–96]	77 [51–91]	89*	90 [82–95]
Benzathine benzyl penicillin injection	69 [58–79]	56 [35–75]	67*	68 [58–77]
Amoxicillin or Ampicillin	88 [79–94]	95 [70–99]	89*	89 [80–94]
Gentamicin	78 [68–86]	71 [48–87]	78*	78 [68–85]
Metronidazole	86 [76–92]	92 [68–98]	100*	87 [78–92]
Acyclovir	17 [10–26]	55 [31–76]	78*	21 [14–29]
Albendazole/Mebendazole	86 [76–92]	77 [53–90]	67*	85 [76–91]
**Medicines for EmONC**				
Injectable oxytocin	98 [91–99]	100 [100–100]	100*	98 [91–99]
Calcium gluconate	43 [33–54]	69 [45–86]	89*	46 [36–55]
Magnesium sulphate	89 [79–94]	95 [70–99]	100*	89 [81–94]
Diazepam	86 [77–92]	85 [59–96]	100*	86 [78–92]
Corticosteroid (prednisolone/dexamethasone)	82 [72–89]	90 [65–98]	89*	83 [73–90]
Ringer Lactate or NaCL 0.9%	88 [79–94]	100 [100–100]	100*	89 [81–94]
**Vaccines**				
Tetanus toxoid vaccine	96 [89–99]	100**	50*	96 [89–99]
BCG vaccine	92 [84–97]	83 [57–95]	89*	92 [84–96]
Vaccine against measles	96 [89–99]	75 [50–90]	22*	93 [88–97]
Oral polio vaccine	99 [92–100]	80 [55–93]	67*	97 [93–99]
Penta vaccine	99 [92–100]	72 [48–88]	11*	95 [91–98]
Vaccine against Rotavirus	99 [92–100]	75 [50–90]	11*	96 [92–98]
Vaccine against pneumococcus	93 [85–97]	70 [46–87]	11*	90 [83–94]
Vaccine against meningococcus	54 [43–64]	50 [29–71]	11*	53 [43–62]

*No CI provided as statistics are without error and reflect true value (all existent MCH nationwide are included in the sample)

** Sample size did not allow generation of 95% CIs, due to healthcare facilities reporting the service was not applicable to their facility

N = number of facilities

IHC = Integrated Health Centres, DH = District Hospitals, MCH = Mother and Child Hospitals, HIV = Human Immunodeficiency Virus, TB = Tuberculosis, ARV = Antiretroviral drugs, HRZE = Isoniazid + Rifampin + Pyrazinamide + Ethambutol quadritherapy, ACT = Artemisin-based Combination Therapy, BCG = Bacillus Calmette-Guerin, EmONC = Emergency Obstetric and Newborn Care

There was overall good availability of drugs for Emergency Obstetric and Newborn Care (EmONC): oxytocin (98% [91–99]), anticonvulsants (89% (81–94] and corticosteroids (83% [73–90]) were widely available, while calcium gluconate was rarer (46% [36–55]) in IHCs (see Table 2). Vaccines were widely available across IHC where immunisation services are primarily provided, with all but meningococcus vaccination present at a level of over 90%. At higher level facilities their availability was lower and more varied, with availability at DH with seven to eight facilities in ten, decreasing to just over 10% for some vaccines at MCH (Table 2).

### Staffing levels and competencies

**Fig 1:**
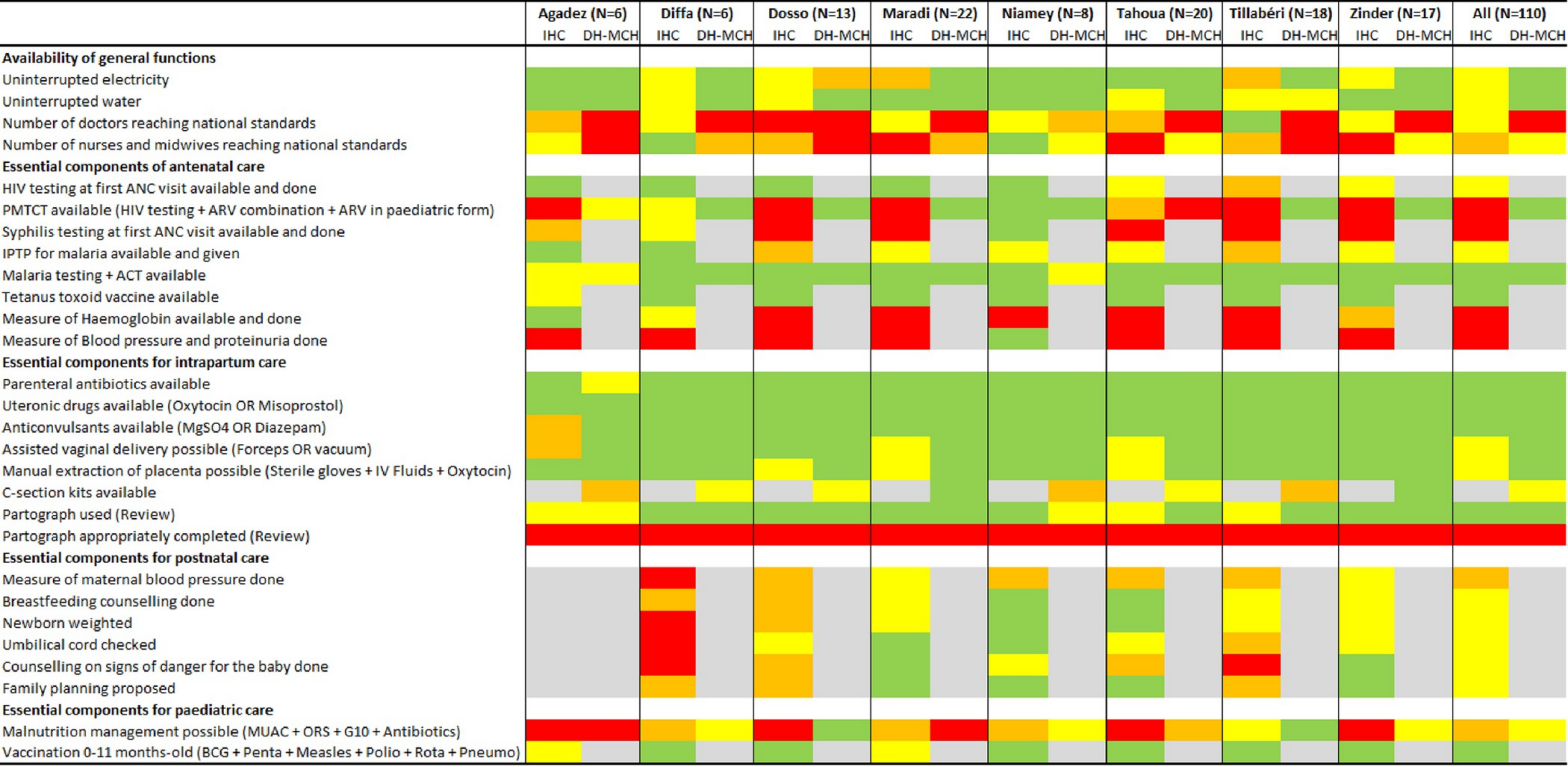
Dashboard for quality of maternal, newborn and child care in Niger.

Staffing levels according to national standards by type of facilities can be found in the dashboard (Fig 1) and show that in most regions, less than half the healthcare facilities had appropriate levels of doctors, nurses or midwives.

Knowledge test topics covered the scope of the services assessed, that is antenatal, intrapartum (incl. emergency obstetric and newborn care), postnatal (mother and baby) and paediatric care, including infective diseases (including syphilis, HIV, TB and malaria) and immunisation. The mean knowledge test score was 45%, with IHC HCPs scoring 42% in Type I facilities and 45% in Type II facilities, HCPs in DH scored on average 47%, while in the top-level facilities the average score was over 50%. The majority of respondents were nurses (247/369) and midwives (98/369), and these cadres scored on average 44% and 49%, respectively. Details of the results can be found in S3 Table.

### Provision of services

Detailed data for this section can be sound in S4 Table (Health facility summary statistics), S5 Table (Exit interviews) and S6 Table (Observations), which also include additional results from the relevant assessment parts. Selected variables are presented to offer a snapshot of the triangulation offered using the three components of the tool in Table 3.

**Table 3:** Triangulation of percentage of clients receiving services in ANC and PNC, by type of data collection (Selected variables) (95% CI).

Facility register records (March 2018)		Observations		Client interviews	
(n = total number of clients recorded in relevant register)		(n = total number of visits observed)		(n = total number of exit interviews incl. clinic card review)	
** **	** **	**Antenatal care**		** **	
Measure of blood pressure (not reported in registers)	NA	HCP took the client’s blood pressure (n = 250)	76 [60–88]	HCP took the client’s blood pressure (n = 404)	84 [76–89]
Measure of proteinuria (not reported in registers)	NA	HCP checked the client’s urine for protein (n = 250)	17 [9–30]	HCP checked the client’s urine for protein (n = 404)	33 [24–42]
Women who had their haemoglobin measured at ANC1 (n = 7,745)	15 [9–24]	HCP took blood to test for haemoglobin (n = 250)	22 [12–36]	HCP took blood to test for haemoglobin (n = 404)	21 [14–29]
Women tested HIV at ANC1 (n = 7,745)	69 [59–77]	HCP tested the client for HIV at ANC1 (n = 92)	62 [48–74)	HCP tested the client for HIV at ANC1 (n = 123)	63 [54–70]
HIV+ women treated at ANC1 (n = 19)	100 [100–100]	Treatment for HIV (given outside ANC room)	NA	Treatment for HIV (given outside ANC room)	NA
Women tested for syphilis at ANC1* (n = 7,502)	15 [10–23]	HCP tested the client for syphilis at ANC1* (n = 70)	17 [10–27]	HCP tested the client for syphilis at ANC* (n = 87)	16 [10–25]
Women with syphilis treated at ANC1 (n = 20)	100 [100–100]	Treatment for syphilis (given outside ANC room)	NA	Treatment for syphilis (given outside ANC room)	NA
Cases of suspect TB (not reported in registers)	NA	HCP asked client if they experienced cough (n = 250)	14 [6–27]	HCP asked client if they experienced cough (n = 404)	31 [25–37]
Women tested for TB at ANC1* (n = 7,502)	0 [0–0]	TB test and referral (not observed)	NA	HCP tested or referred for TB (n = 310)	4 [1–10]
Eligible women receiving IPTp in any ANC visit (n = 12,205)	94 [88–97]	HCP gave IPTp and explained why (n = 250)	75 [62–85]	HCP gave IPTp and explained why when eligible (n = 379)	76 [68–83]
Cases of suspect malaria (not reported in registers)	NA	HCP took the client’s temperature	13 [5–30]	HCP took the client’s temperature (n = 404)	31 [25–38]
Women tested for malaria at ANC1 (n = 7,745)	19 [12–28]	HCP tested for malaria (n = 250)	24 [13–40]	HCP tested for malaria (n = 404)	38 [31–46]
Woment with malaria treated at ANC1 (n = 259)	99 [95–100]	Treatment for malaria (given outside ANC room)	NA	Treatment for malaria (given outside ANC room)	NA
Signs of danger in pregnancy (not reported in registers)	NA	HCP discussed signs of danger in pregnancy (n = 250)	63 [51–74]	HCP discussed signs of danger in pregnancy (n = 404)	60 [53–66]
Iron and folic acid supplementation (not reported in registers)	NA	HCP gave iron and folic acid tablets (n = 250)	85 [70–93]	HCP gave iron and folic acid tablets (n = 404)	84 [77–89]
Birth preparation (not reported in registers)	NA	HCP discussed the birth plan with client (n = 250)	50 (38–62]	HCP discussed the birth plan with client (n = 404)	40 [33–48]
Counseling for family planning (not reported in registers)	NA	HCP counselled on family planning (n = 250)	23 [12–39]	HCP counselled on family planning (n = 404)	38 [32–46]
** **	** **	**Postnatal care**		** **	
Women who had their haemoglobin measured in PNC (n = 2,437)	21 [14–29]	HCP took blood to test for haemoglobin (n = 224)	1 [0–5]	HCP took blood to test for haemoglobin (n = 375)	8 [4–15]
Women counseled for family planning in PNC (n = 2,437)	85 [77–90]	HCP counselled on family planning (n = 224)	74 [60–84]	Family planning counseling (not asked in interviews)	NA
Support on breastfeeding (not reported in registers)	NA	HCP checked the client’s breasts (n = 224)	41 [25–59]	HCP counselled on breastfeeding (n = 375)	68 [61–75]
Newborn examination (not reported in registers)	NA	Newborn examination (detailed: See suppl. tables)	NA	HCP examined baby (n = 375)	71 [64–77]
Mother examination (not reported in registers)	NA	HCP measured uterine height (n = 224)	27 [14–46]	HCP checked uterus for involution (n = 375)	26 [19–35]
Women tested for HIV (not reported in registers)	NA	HCP tested for HIV (n = 224)	15 [7–27]	HCP noted HIV status in clinic card (n = 375)	40 [32–50]

*Calculated for facilities where given service was reported as offered

### Uptake of MNH services

Review of facility registers and records for attendance shows that in March 2018, 22,301 women attended for the ANC visit, of which the great majority at IHC (21,043). Of those 7,745 (7,502 at IHC) attended for their first (booking ANC), while 4,469 (4,282 in IHC) attended for their fourth or later visit. A total of 6,225 women gave birth in facilities, of which 2,526 were at IHC, 1,654 at DH and 2,045 at MCH facilities. Postnatal services were attended by 1,883 women, of which the majority (1,614) in IHC. Among children in outpatient paediatric services, 39,781 under-5s attended an outpatient clinic. In inpatient paediatric services, 7,961 children were admitted, of which the majority in higher level facilities (6,426 in DH and 1,203 in MCH), with only one IHC in the sample providing the service.

### Screening during antenatal care

Based on observation, screening for preeclampsia was incomplete in ANC services, as 76% [60–88] women had their blood pressure measured, with proportion of those tested remaining stable even when checked for equipment availability (78% women had their blood pressure measured raising to 84% where BP machine was present within the sampled facilities). However, proteinuria tests were done for only 17% [9–30], which remained at a similar level even adjusting the calculation for facilities which had the necessary equipment and/or supplies (23% for all respondents, compared to 26% in facilities with dipsticks). Of note, only half, 49% [35–63], of HCP were observed explaining the BP measurement to their clients. Exit interviews provide similar findings for BP measurement reported by 84% [76–89], while proteinuria tests were done more frequently, although still not often (33% [24–42]).

Screening for anaemia was also inconsistently observed. Haemoglobin tests were observed for 22% [12–36] clients and 82% [68–90] had their conjunctiva checked. In exit interviews, 21% [14–29] clients reported having had their haemoglobin tested.

In regard to infectious diseases, despite the overall fair availability of means for screening at most levels and treatments in referral centres, register data showed that only 69% [59–77] of women were tested for HIV and 15% [10–23] for syphilis during their first ANC visit (ANC1) and that 0% [0;0] of women were screened for TB. Women reported being provided with and explained about intermittent preventive treatment in pregnancy (IPTp) in 98% [95–99] of exit interviews.

Data from the observation of consultations showed fairly similar results to register data, with 62% [48–74] of women tested for HIV and 17% [10–27] in ANC1. Temperature was measured in 13% [5–30] of observed consultations. However, provision of intermittent preventive treatment in pregnancy (IPTp) from second trimester onwards was observed less frequently (75% [62–85]) than reported in exit interviews. Clinical signs for TB were not routinely looked for, with healthcare providers inquiring about cough in 14% [6–27] and night fever in 32% [22–45] of cases.

Nearly all women had their fundal height measured (97% [94–99] and 88% [76–94] had their baby’s heartbeat listened to. Eighty-five percent [70–93] were prescribed with or given iron tablets and TT vaccine was given to 58% [47–67]. Two-thirds were advised about having a skilled birth attendant for childbirth (66% [55–76]) and 63% [51–74] were informed about danger signs.

Exit interviews findings were consistent with observed practice with 95% [90–97] women reporting their fundal height measured and fetal heartbeat listened to (83% [75–88]), 84% [77–89] having received iron tablets and 79% [73–83] TT vaccination. Similarly, 60% [53–66] remembered receiving guidance on danger signs.

### Intrapartum services

In intrapartum services, based on exit interviews, 71% [64–78] were reported to move and change positions as desired during labour, but only around half were offered pain relief (46% [39–53]), encouraged to breastfeed with an hour of giving birth (55% [48–63]) and were informed about exclusive breastfeeding (49% [42–56]). Most babies were reported to be dried and wrapped immediately after birth (86% [80–90]), weighed (89% [82–93]) and placed immediately skin-to-skin (75% [69–81]). However, only 7% [4–11] babies received an identification tag.

### Partograph usage and quality

During labour, partographs were often used (86% [80–91%] of deliveries) but filled poorly, as assessed in the partograph review. Individual sections were inconsistently completed, with for instance only 68% [60–75] of all partograph reviewed displaying records of uterine contractions, 70% [62–76] providing at least one record of cervical dilatation, 79% [72–84] at least one of maternal blood pressure and 83% [76–88] at least one of fatal heart rate (see details on completion of partograph sections in S7 Table).

### Assessment of the mother in PNC

Observation of PNC services showed 49% [33–65] of women had their BP measured, 15 [0–5] haemoglobin tested and 63% [46–76] had their conjunctiva checked. Uterine height measurement, examination of breasts and examination for lochia were at 27% [14–46], 41% [25–59], and 51% [36–66], respectively. Results pertaining screening for high blood pressure, and anaemia, as well as clinical examination from exit interviews were consistent, with 51% [43–60] had their BP measured, 8% [4–15] had their haemoglobin tested. Infectious disease screening of mothers was rarely observed, with 15% [7–27] tested for HIV and 4% [0;11] for malaria. Examination of uterus was reported by 26% [19–35].

### Assessment of the baby in PNC

Comprehensive assessment of the baby was not frequently observed; with 65% [47–80] weighed, 35% [22–52] had their heartbeat listened to and 45% [30–60] their stomach palpated and under 30% (28% [16–45] had their length measured. Only 29% [18–44] had their growth cart filled. Again, some results from observation were sustained in exit interviews: Clients reported their babies being weighed in 70% [62–77] of cases and length measured in 47% [39–56] of them. Eighty-one percent [73–87] babies had their polio and 77% [68–84] BCG vaccinations provided at PNC.

### Paediatric services

In paediatric outpatient clinics, one in five children suffered from malnutrition. Out of 0-11months’ olds eligible for immunisation between under half and almost two-thirds received them during their facility visit (polio vaccination uptake was highest at 61% [50–71]). Over three-quarter of children had their temperature measured 78% [72–83} and nearly all left with a medication (96% [94–98]). Among under-5s, 37% [26–50] were admitted to inpatient clinics due to severe acute malnutrition, 26% [18–36] due to malaria, 19% [12–29] due to pneumonia and 13% [10–17] with diarrhoea.

### Experience of care

Exit interviews and observations provided insight into Respectful Maternity Care (RMC). Between the sources of data, it showed that clients were greeted (ANC at 93% [89–96], Intrapartum at 89% [84–92], PNC at 93% [85–97] and Paediatrics at 93% [89–95]), women felt their privacy was respected (ANC at 98% [95–99], Intrapartum at 98% [95–99], PNC at 89% [83–94] and Paediatrics at 94% [89–96]) and were not shouted at (ANC at % 95% [92–97], Intrapartum at 95% [92–96], PNC 96% [92–98] and Paediatrics at 95% [91–97]. While the majority felt they could discuss concerns (ANC at 65% [57–71], Intrapartum at 53% [45–60], PNC at 56% [48–64], and Paediatrics at 47% [40–55])fewer were encouraged to ask questions (ANC at 22% [16–29], 10% [7–15] at Intrapartum, 25% [20–32] at PNC and 25% [20–31] in Paediatrics). Seeking permission before examination and explaining results were scored lower and varied between services: ANC seeing permission at 57% [50–64] and explaining results at 59% [50–67]; Intrapartum 37% [30–45] and 30% [22–39]; PNC at 39% [32–46] and 9% [4–22] and Paediatrics at 44% [38–50] and 31% [24–39], respectively.

Observation data showed similarly high levels of RMC with over nine in ten clients being greeted (ANC at 97% [91–99] and PNC at 93% [85–97]) and eight in ten being asked about concerns (ANC at 83% [72–90] and PNC at 84% [76–90]). Findings can be found in S5 and S6 Tables.

### Waiting times and overall times spent in facility

Prior to conducting observation, HCP being asked for the overall times spent in facility reported women spent on average 105 minutes (ranging from 30 to 299) for ANC in IHC, and, 163 minutes in total (110 to 291) in higher level facilities (DH and MCH). Women attending for PNC in IHC spent on average 66 minutes (ranging from 27 to 159) in the facility, and in higher level facilities (DH and MCH), the timings were 111 minutes on average (32 to 230).

Based on exit interviews, waiting times were a concern at ANC where 29% [23–36] felt they did not have to wait too long for services, waiting times were a concern for half of client’s services at PNC 51% [45–57]. A further 60% attending paediatric services reported not having to wait too long.

The summary dashboard displaying the availability and provision of key components across the continuum of care is shown in Fig 1. This is a composite presentation of structure and process indicators across the assessment areas. It shows a patchy picture, reflective of structural gaps (lack of equipment, supplies and medicines), as well as incomplete provision of services which are in principle available, thus flagging opportunities of improvement over the quality of care provided across all regions.

## Discussion

This comprehensive review of different aspects of the quality of care in a nationally representative sample of healthcare facilities in Niger in 2018, was conducted using a tool specifically designed for assessing QoC across the full continuum care in MNH.

Overall, more than a quarter of healthcare facilities were impaired by discontinuous electricity or water supply, and less than half had adequate staffing levels according to national standards. Competencies of HCPs showed gaps, which potentially hinder the quality of care they can offer. Routine screening for infectious diseases was incompletely integrated at ANC1 (booking) visit. Opportunities for improvement of integrated care were observed regarding prevention, screening and management of malaria, preeclampsia, anaemia, and tuberculosis. In most instances, low availability of supplies seemed to impact the delivery of care but was not the sole factor affecting care provision. At delivery stages, the inconsistent appropriate completion of partographs showed that despite the fair availability of EmONC essential drugs and supplies, improvement was required in the care provided. Finally, there was variable levels of respectful care provided across the continuum of care, with gaps mainly concerning challenges on the communication and softer skills rather than perpetuating physical abuse, in line with other studies in sub-Saharan Africa [28].

### Limitations

There were several challenges in the development of the QoC tool and study. Firstly, the information collected on infrastructure, equipment and supplies was not a precise assessment of functionality of referral systems or stock management, which would have made the data collection more intensive and risked respondent fatigue. Secondly, the observations or record review did not assess the quality of the clinical examination nor the accuracy of diagnosis, although the tool was able to measure the level of completeness of the service provision. Slightly higher results on indicators retrieved via observation may be indicative of Hawthorne effect [29], though equally there could be an issue with recall for post-service interviews. However, combining sources of data allowed for a fuller picture to be built and minimise the risk of over or under reporting relevant findings. Parts of the assessment tool were quite limited, such as the paediatric care, which was focused on the neonatal and then under-5 period. Thirdly, interpretation of findings for screening of diseases such as malaria or TB which is based on clinical, biological or radiographic examination was limited in our tool. Regarding TB for instance, our findings show that enquiring for cough was not done routinely which suggest missed opportunities, as the 2017 Nigerien national TB control program technical guide gives the definition of suspected cases as patients with a cough >2 weeks, with or without haemoptysis, thoracic pain, weight loss, fever or night sweats. However, data collectors were not all from a medical background that would have allowed them to quickly and reliably assess if a client indeed was suspect cases of TB or malaria at time of observations, which renders the interpretation of the proportion of them getting tested difficult [30]. Fourthly, data collection was undertaken in one reference month only and thus does not account for the seasonality of disease incidence and client uptake patterns. However, collecting data over one month was deemed appropriate to the study team due to resources and the difficulty of fieldwork conditions. Finally, no systematic information on costs was collected, although this is likely an important determinant of access to care in Niger: indeed, maternal mortality ratio had decreased substantially from 715 in 2006 to 535 per 100,000 live births in 2012 due partly to a fee exemption policy for ANC, family planning and caesarean section implemented since 2006 for pregnant women [5].

### Recommendations for quality improvement of MNH care in Niger

Several ways for improving quality of care in MNH in Niger could be explored. The first gap identified is the need for targeted improvement of supplies for essential components across the continuum of care, such as syphilis or HIV-syphilis dual rapid diagnostic tests, reliable Haemoglobin measurements, and means for malaria prevention among mothers and babies [31–34]. Supply shortage did not seem to be the only factor impeding delivery of care. An example can be seen with the rarity of haemoglobin testing: although seemingly linked to low availability of testing means at IHCs, the low testing rates persisted at MCHs despite all MCHs having the capacity to test. Thus, a second recommendation would be to combine increase in supplies with better integration of services, using models like integrated point-of care testing (POCT) for increased efficiency of antenatal and postnatal services [31]. Although the long waiting times identified for ANC and PNC visits in this study point at difficult working conditions for HCPs which may hinder such improvements, there is evidence that an initial increase in workload from focused service reorganization can decline later with enhanced efficiency in providing additional services [35, 36]. In cases where POCT at primary level would not adequately align with national guidelines recommending referral to a higher facility, there is a case for capacity strengthening of clinical screening of conditions such as tuberculosis which can have high prevalence among both HIV-seropositive and seronegative pregnant women [37]. Moreover, considering the dwindling ANC attendance, childbirth happening mainly outside of healthcare facilities and poor PNC attendance, the case is strong for providing as many relevant services as possible whenever women come in, building on the client contact at ANC1 to provide support and guidance to pregnant women. A holistic approach exploring reasons for non-completion or late occurrence of first ANC contact during pregnancies would be warranted to address the limited uptake of services and complement the quality improvement opportunities identified at facility level and presented in this paper. Given the findings from this assessment are consistent with the SDI report, which showed just over a quarter of HCPs were able to correctly provide diagnostic accuracy (27%), while 17% showed adherence to clinical guidelines and only 12% were able to correctly manage maternal and newborn complications [38] a further recommendation from the incomplete delivery of essential services found in the study would be to strengthen HCP capacity through competency-based training, mentoring and quality improvement methods, based on recommended standards of care across the MNH continuum of care [9]. Integral to this effort is the important opportunity for improvement in respectful care. Its assessment from the study revealed more courteous attitudes (greeting, no shouting) compared to a similar study by Moyer et al. [39, 40] but evidenced a common pattern across the continuum of care: Many women reported HCPs did not actively explain results, inform on diagnosis or ensure provision of appropriate support for women and babies. Besides, caution over the low rate of abuse reported by service users is warranted, as it was documented that women seeking care may consider respectful dialogue as a luxury rather than an expectation of good services received [41]. The findings are consistent with other studies conducted in West African countries where theoretical respectful attitudes fail to translate into practice and call for the strengthening of soft skills alongside clinical practice [39, 42].

### Lessons learned for assessment of QoC in MNH

This study offers lessons learned which could apply to other QoC assessments in MNH. The inclusion of multiple perspectives contributed to the internal validation of the tool: The good consistency of results between registers, exit interviews and observations provided triangulation and a good level of confidence in the findings. Assessing QoC offers challenges to balance comprehensiveness and manageability. The tool was innovative in its inclusion of the full continuum of care, incorporating the quality of ANC and PNC as an important determinant of maternal and neonatal mortality and morbidity [12]. The in-depth assessment of the quality of ANC and PNC through register review, interviews and observation, enabled identification of gaps in the early detection of pregnancy complications and in the integration of infectious disease control during and after pregnancy, such as PMTCT (prevention of mother to child transmission for HIV) and malaria prevention. Of note, the total number of observations of ANC and PNC were lower than the initially planned targets across facility levels because these services were rarely provided in DHs and MCHs, but it still offered a good overview for primary health services at IHC facilities. Overall, the tool offered an understanding of gaps in QoC at each step of the full continuum of care from the perspective of facility in-charges, HCPs and users in Niger. Another important aspect of assessment of QoC in MNH is the capture of the experience of care (32). The tool integrated indicators drawn from reviews of qualitative evidence of what constitutes respectful care for women. Key domains, such as being free from mistreatment, privacy, communication and information, consent seeking, or right to companionship, allowed the study to offer an objective snapshot of the state of RMC in Niger [40, 43]. This component also accounted for questions related to satisfaction (e.g. acceptability of time spent at facility) to balance the findings with user’s subjectivity and reinforce the person-centred perspective of the findings [44]. Notably, the study did not expand on aspects of care that influence social representations of respect, shame and human interactions that would in turn define the acceptability of care perceived by women and communities [45, 46]. However, by involving MNCH relevant stakeholders in its review, the tool was tailored to include standards of care from national policies as well as international ones, and respectful maternity care indicators were reviewed to ensure we were assessing quality of care in a culturally sensitive manner. Systematic reviews of RMC literature show a degree of diversity in the available tools, but the main domains are aligned, and the tool used by LSTM falls within those [43, 47]. While previous WHO tool used in Malawi was reported as time consuming to administer [16], by focusing on essential and agreed indicators, it was possible to visit 110 healthcare facilities in six weeks to collect information from more than 2500 pregnant women and mothers, and healthcare providers, with travels covering 15,000km. Finally, the use of a dashboard in displaying findings provided a useful and appreciated picture of thematic priorities in MNH in Niger that can be used by decision-makers and be replicated in similar settings. Subsequently to the implementation of this research, a study by Brizuela et al. has shown that when benchmarked against the WHO generic framework for the quality of maternal and newborn health care, existing tools only partially covered quality dimensions, despite requiring substantial resources to be implemented [48]. Challenges with developing standardised tools and indicators to be used globally within maternal care assessments have been well identified [49, 50], but since conducting the assessment great strides have been made in incorporating and standardising quality of care measurements in the established tools, which at the time of the survey were not as common. This includes the evolution of the WHO SARA tool into the Harmonised Health Facility Survey [51], updates to the SPA [52] and SDI [14] tools provide strong foundations for more in-depth assessment of MNCH care, including quality. Further improvement to assessment tools for QoC in MNCH could include more in-depth assessments of community-led and based support systems, as well as of the organisation and management of care. More indicators could be added to provide recommendations to ministries and managers, as to how to bring efficiency in the integration of care, reimbursement process, communication in referral and data use for improving quality of care in MNH.

## Supporting information

S1 Checklist Strobe checklist. (PDF)

S2 Checklist Inclusivity in global research. (DOCX)

S1 Table Overview of the QoC assessment tool components and numbers of responses targeted. (TIFF)

S2 Table Overview of data collected. (TIFF)

S3 Table Percentage of correct responses for selected aspects of knowledge assessment. (TIFF)

S4 Table Health facility summary statistics on uptake and outcomes (Total number and 95% CI). (TIFF)

S5 Table Exit interviews–selected results for ANC, Intrapartum, PNC and Paediatric services (95% CI). (TIFF)

S6 Table Observations–selected results for ANC and PNC services (95% CI). (TIFF)

S7 Table Use and completion of partograph reviews showing percentage (95% CI)s of partographs that displayed completion of the relevant section. (TIFF)
