# Supplementary material for: Cross sectional survey of maternal and newborn quality of care in Niger: Selected findings, lessons learned and recommendations
Source: PLOS Glob Public Health. 2024 Nov 13;4(11):e0003268. doi: 10.1371/journal.pgph.0003268 (PMC11559989; doi:10.1371/journal.pgph.0003268)
Supplement: S2 Checklist — (DOCX) [file pgph.0003268.s002.docx]

Inclusivity in global research

PLOS’ policy on inclusivity in global research aims to improve transparency in the reporting of research performed outside of researchers’ own country or community and ensures that PLOS publications reporting global research adhere to high standards for research ethics and authorship. Authors of relevant research articles may be asked to complete the questionnaire below, which outlines ethical, cultural, and scientific considerations specific to inclusivity in global research. This questionnaire may be requested when researchers have travelled to a different country to conduct research, if research uses samples collected in another country, research with Indigenous populations or their lands, or if research is on cultural artefacts. Researchers travelling to another country solely to use laboratory equipment will not normally be required to complete the questionnaire. However, the questionnaire can be requested at the journal’s discretion for any submission – if you have been requested to complete this questionnaire by the PLOS journal you submitted to, please do so.

Please complete the questionnaire below and include this as a Supporting Information file with your manuscript. Note that if your paper is accepted for publication, this checklist will be published with your article in the supporting information files. Please ensure that you reference the checklist in the main body of your manuscript. We suggest adding a subsection ‘Inclusivity in global research’ to your Methods section and adding the following sentence: “Additional information regarding the ethical, cultural, and scientific considerations specific to inclusivity in global research is included in the Supporting Information (SX Checklist)”

The questions have been designed to be applicable to a wide range of study types, and there are subsections for both human subjects research and non-human subjects research. If any of the questions are not relevant to your research please mark them as “N/A” as appropriate.

**Ethical considerations, permits and authorship**

*This section is applicable to all research types.*

Provide details as to who granted permissions and/or consent for the study to take place in the Methods section of your manuscript. This should include the names of **all** ethics boards, governmental organizations, community leaders or other bodies that provided approval for the study. If individuals provided approval refer to these people by their role or title but do not list their name(s).

Reported on pages number: 6, 8

If there were any deviations from the study protocol after approval was obtained please provide details of these changes in the Methods section of your manuscript.
Did this study involve local collaborators that are residents of the country where the research was conducted or members of the community studied? If you do not have any authors from said communities, please provide an explanation for this below.

Reported on page number: NA

Authors Aminata Tinni Konate, Ibrahim Souley, Lucien Omar Marcel are resident of the country where the research was conducted. At the time of the study Ibrahim Souley was General Director of Reproductive Health, and Lucien Omar Marcel was Director for Mother and Child Health, both at the Ministry of Public Health. Amninata Tinni Konate was technical advisor to the Ministry.

The Ministry delegated field work activities to the National Institute of Statistics (INS), for which collaborators are acknowledged in lines 462-464 (page 21) of the manuscript. The INS hired data collectors, all Nigerien nationals, for conducting data collection activities. Team leaders are listed and thanked in lines 480-483 (page 21) of the manuscript. Their role, training and activities are described in the methods section.

Everyone listed as an author should meet PLOS’ criteria for authorship and all individuals who meet these criteria should be included in the author byline, rather than the acknowledgements. For further information please see the journal’s Authorship Policy.

**Human subjects research (e.g. health research, medical research, cross-cultural psychology)**

Did you obtain written informed consent from a representative of the local community or region before the research took place? How did you establish who speaks for the community? Details of written informed consent obtained from study participants should be reported separately in the Methods section of your manuscript.

No community approval was sought as the survey was strictly conducted within healthcare facilities which are under the authority of the Ministry of Public Health, represented in the study team and authors. Written consent was sought from healthcare facility in-charges, healthcare providers for each consultation rooms, as well as any client participating to either observations or interviews. The consent seeking process is described in lines 150-159 (page 6) of the manuscript.

How did members of the local community provide input on the aims of the research investigation, its methodology, and its anticipated outcome(s)?

Stakeholder consultation meetings were done under the leadership of the Ministry of Public Health, with national experts in maternal and newborn health. All participants spoke French and this was the language used. As this was a facility-based survey, no inputs of local communities were sought in the design of the study.

When engaging with the local community, how did you ensure that the informed consent documents and other materials could be understood by local stakeholders?

Will the findings of the research be made available in an understandable format to stakeholders in the community where the study was conducted (e.g. via a presentation, summary report, copies of publications, etc.)? Please provide details of how this will be achieved.

Informed consent documents were reviewed by the Ministry of Public Health and Nigerien National Committee for Health Research (CNERS) for appropriateness and clarity. The process for impartial witnesses is described in lines 150-159 (page 6) of the manuscript. The main purpose of the survey was to provide a clear snapshot of quality of care in maternal and newborn health to policymakers and technical staff at the Ministry of Public Health. Findings were disseminated in a workshop with Nigerien technical stakeholders for Maternal and Newborn Health. As the findings do not focus on any particular community or locality, there was no intended direct feedback to communities or general public by the study team itself, rather the Ministry will be sole decider about further dissemination of the data.

**Non-human subjects research using specimens/ animals collected as part of the study, or those housed in archival collections. Examples include archaeology, paleontology, botany and zoology.**

Did the permission you obtained from a local authority to perform the study include an agreement on access to outputs and benefit sharing? This may include procedures to enable fair distribution of the benefits and resources arising from the research performed. Please include any details of Prior Informed Consent and Benefit Sharing Agreements obtained. These may be required by field-specific regulations, for example the Convention on Biological Diversity (CBD) and the associated Nagoya Protocol.

NA

If the material used in your study was imported, please A) provide the year it was imported and B) indicate whether permits were obtained to import/export the materials used, C) provide details of any permits obtained. If this information is not available, please indicate this.

NA

If you used archival specimens, please state how the material used in your study was acquired by the institute it is held in and provide details of any permits obtained for the original excavations/ sample collection. If this information is not available, please indicate this.

NA

How was the potential cultural significance of the materials collected in your study to local communities considered in your research design? Were Indigenous peoples and/or local researchers and institutions involved with archaeological excavations / collection of specimens? If so, please provide a description of their involvement.

NA

If your manuscript includes photographs of human remains please indicate whether authors obtained permission from descendants or affiliated cultural communities to do so.

NA
