# Supplementary figures and images for: Cross sectional survey of maternal and newborn quality of care in Niger: Selected findings, lessons learned and recommendations
Source: PLOS Glob Public Health. 2024 Nov 13;4(11):e0003268. doi: 10.1371/journal.pgph.0003268 (PMC11559989; doi:10.1371/journal.pgph.0003268)

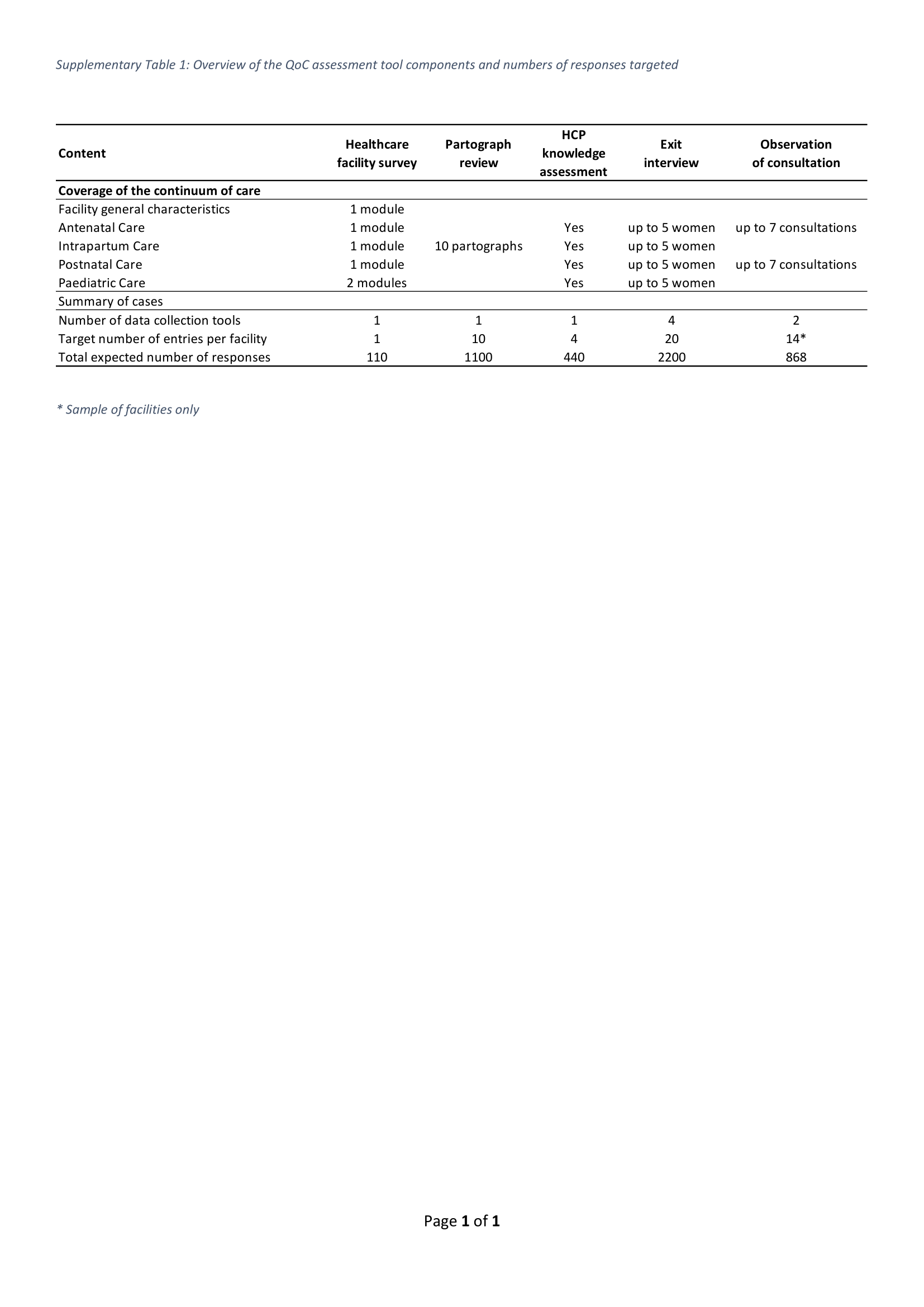

Supplement: S1 Table — (TIFF) [file pgph.0003268.s003.tiff]

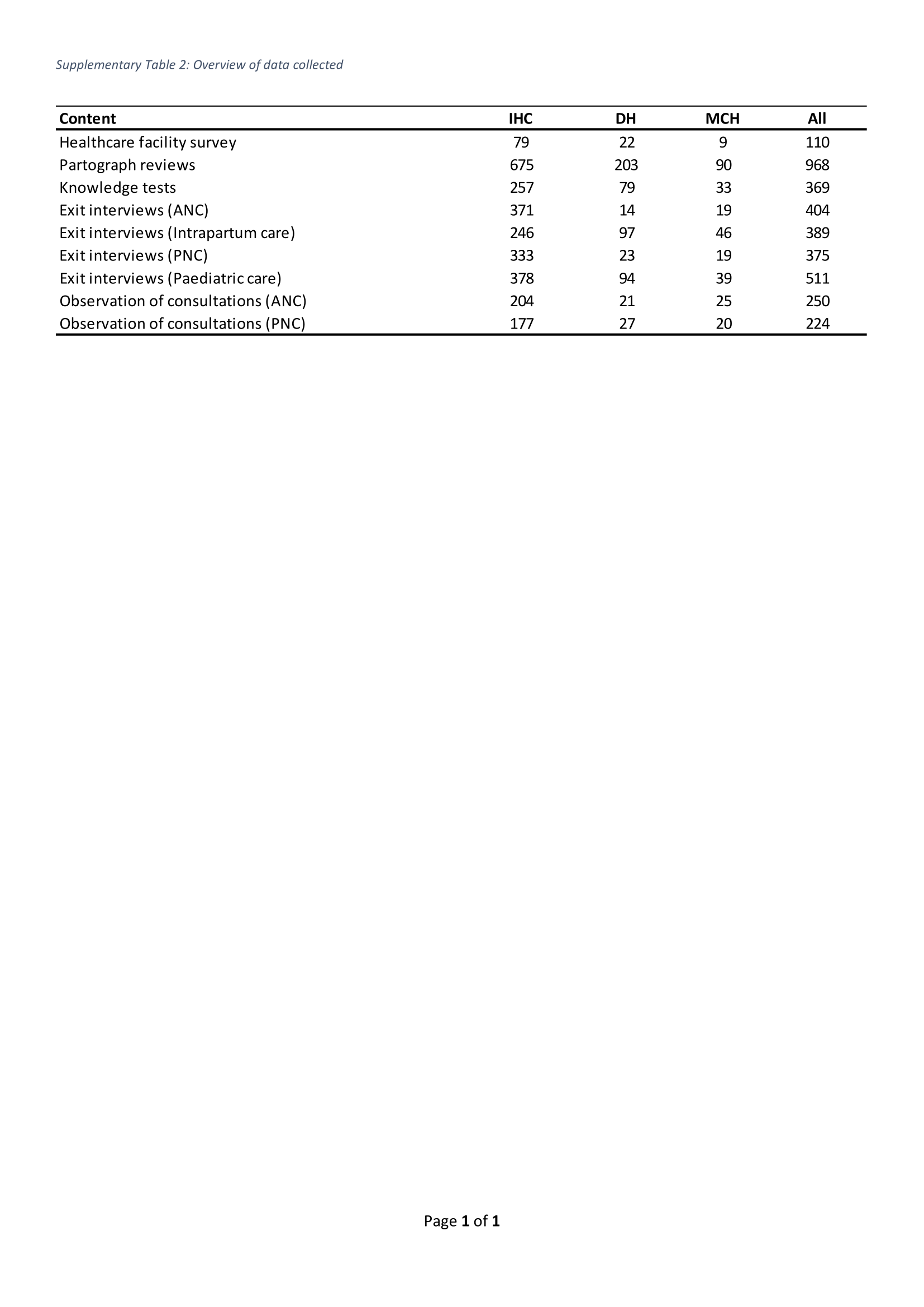

Supplement: S2 Table — (TIFF) [file pgph.0003268.s004.tiff]

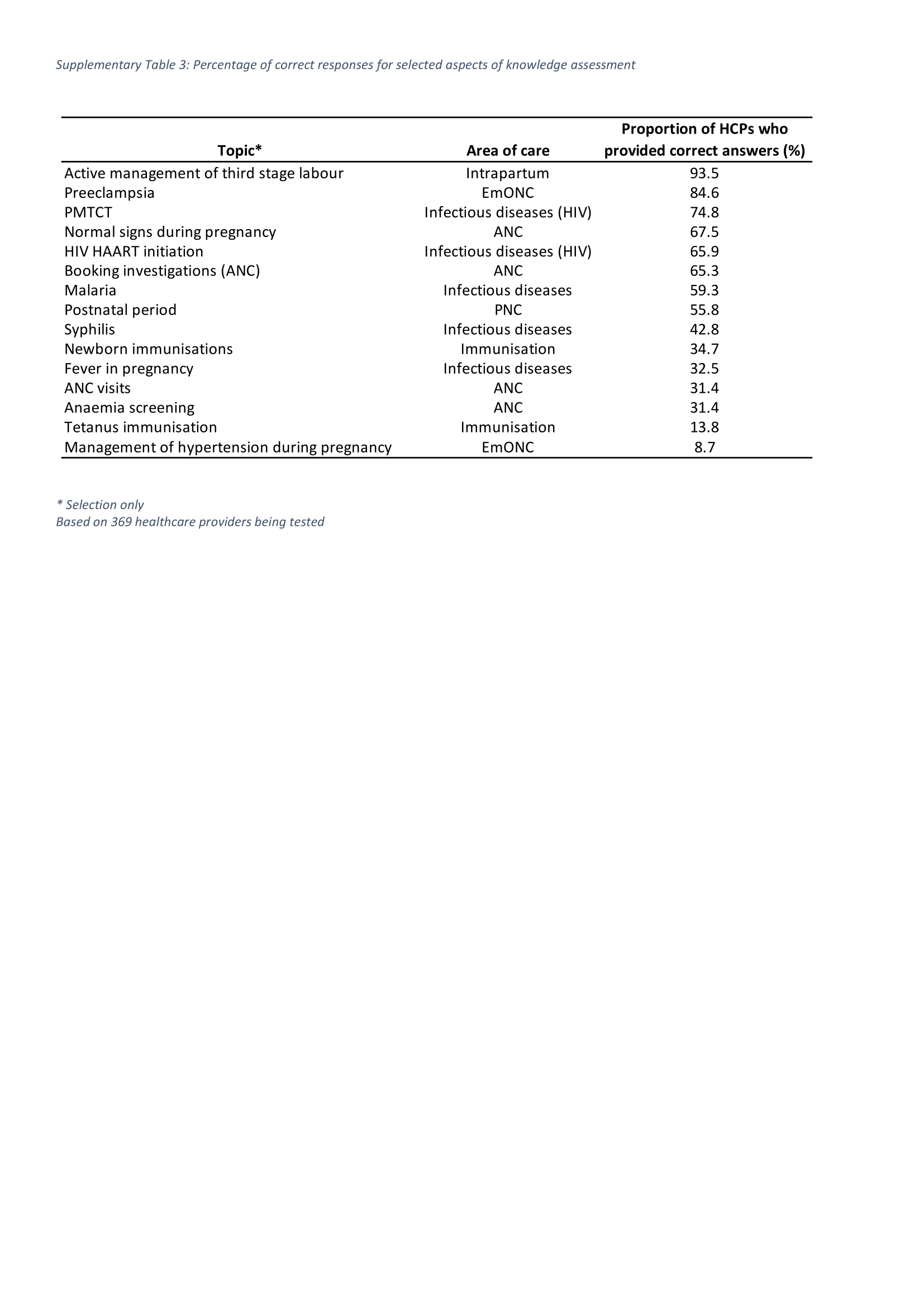

Supplement: S3 Table — (TIFF) [file pgph.0003268.s005.tiff]

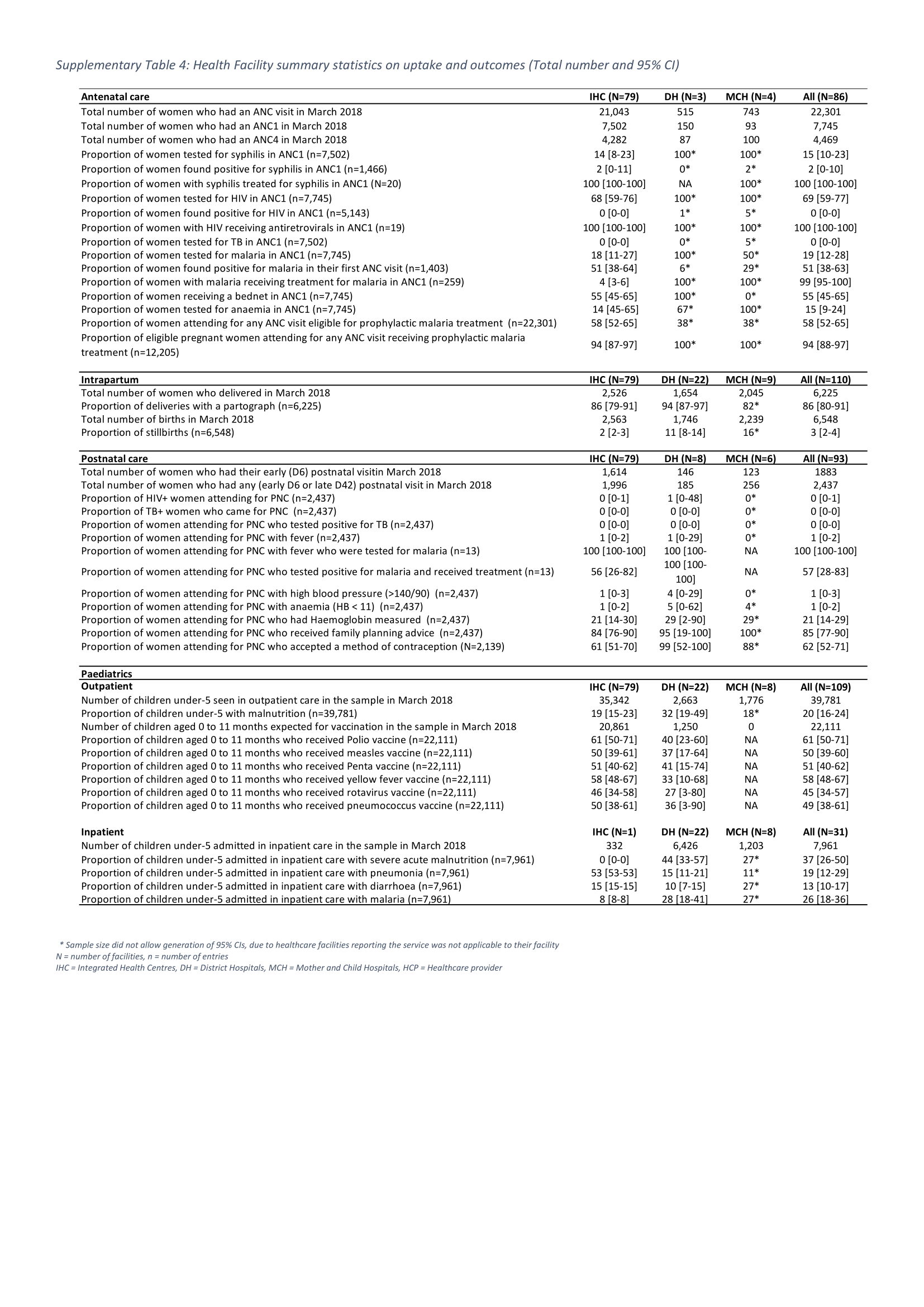

Supplement: S4 Table — (TIFF) [file pgph.0003268.s006.tiff]

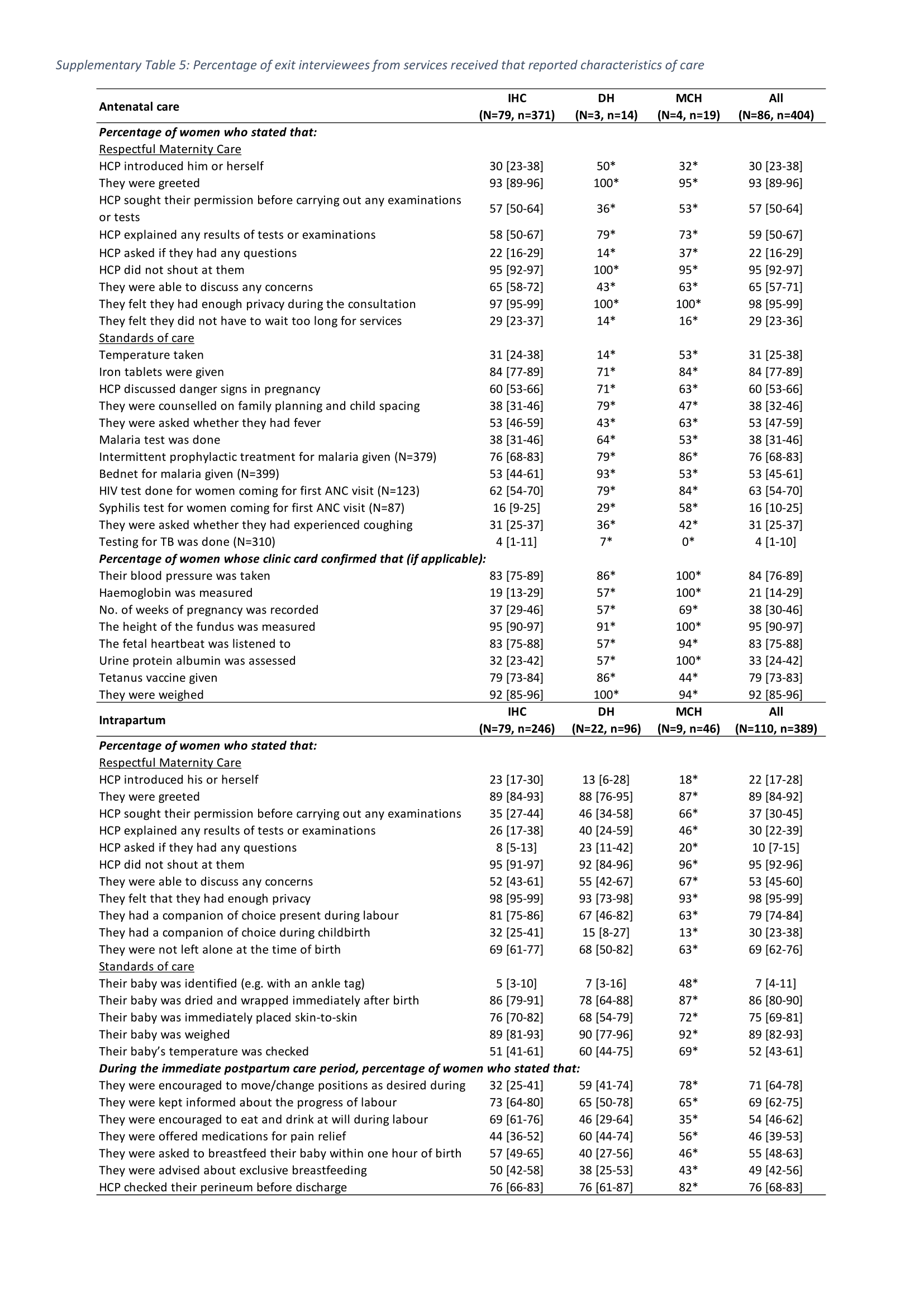

Supplement: S5 Table — (TIFF) [file pgph.0003268.s007.tiff]

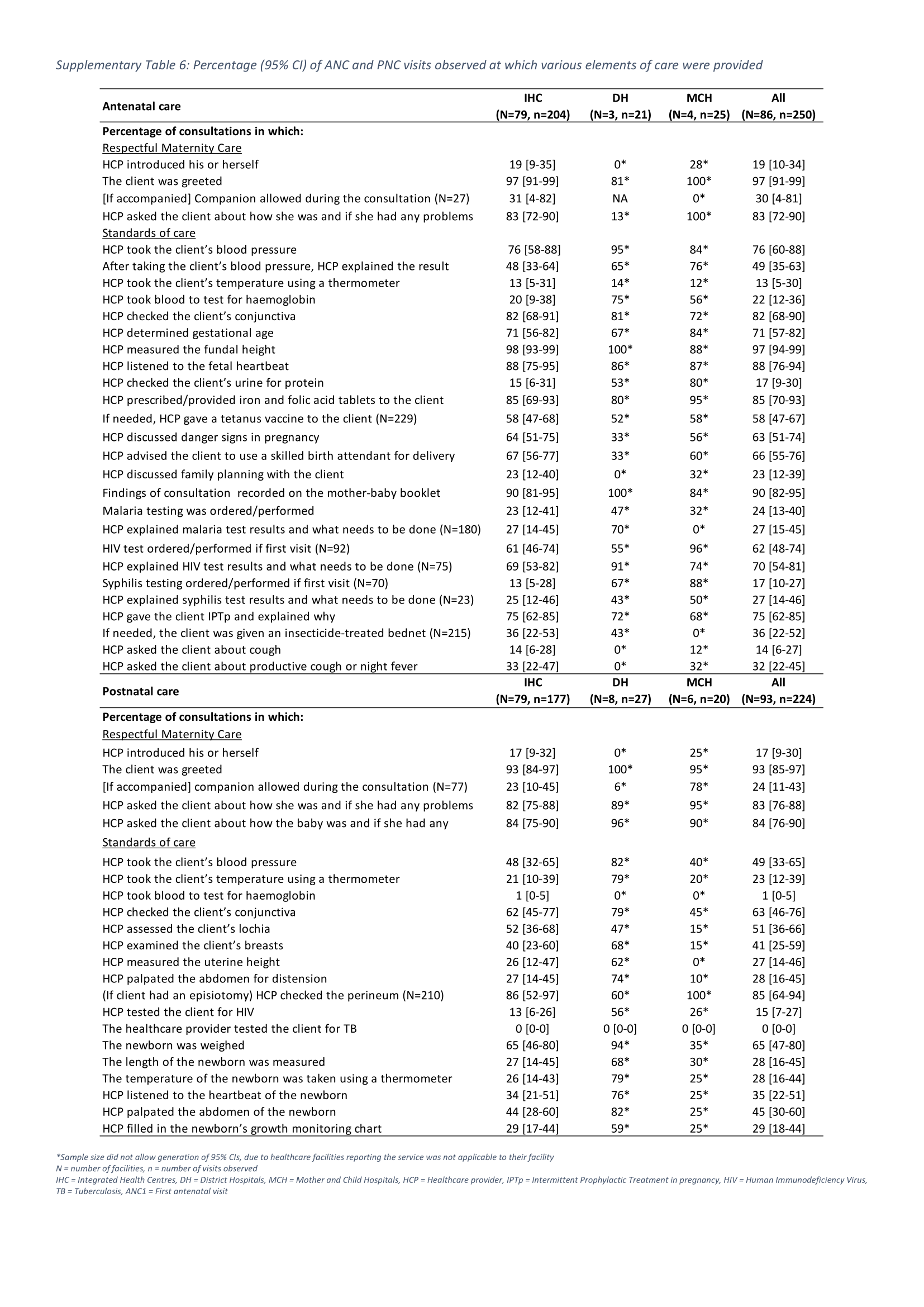

Supplement: S6 Table — (TIFF) [file pgph.0003268.s008.tiff]

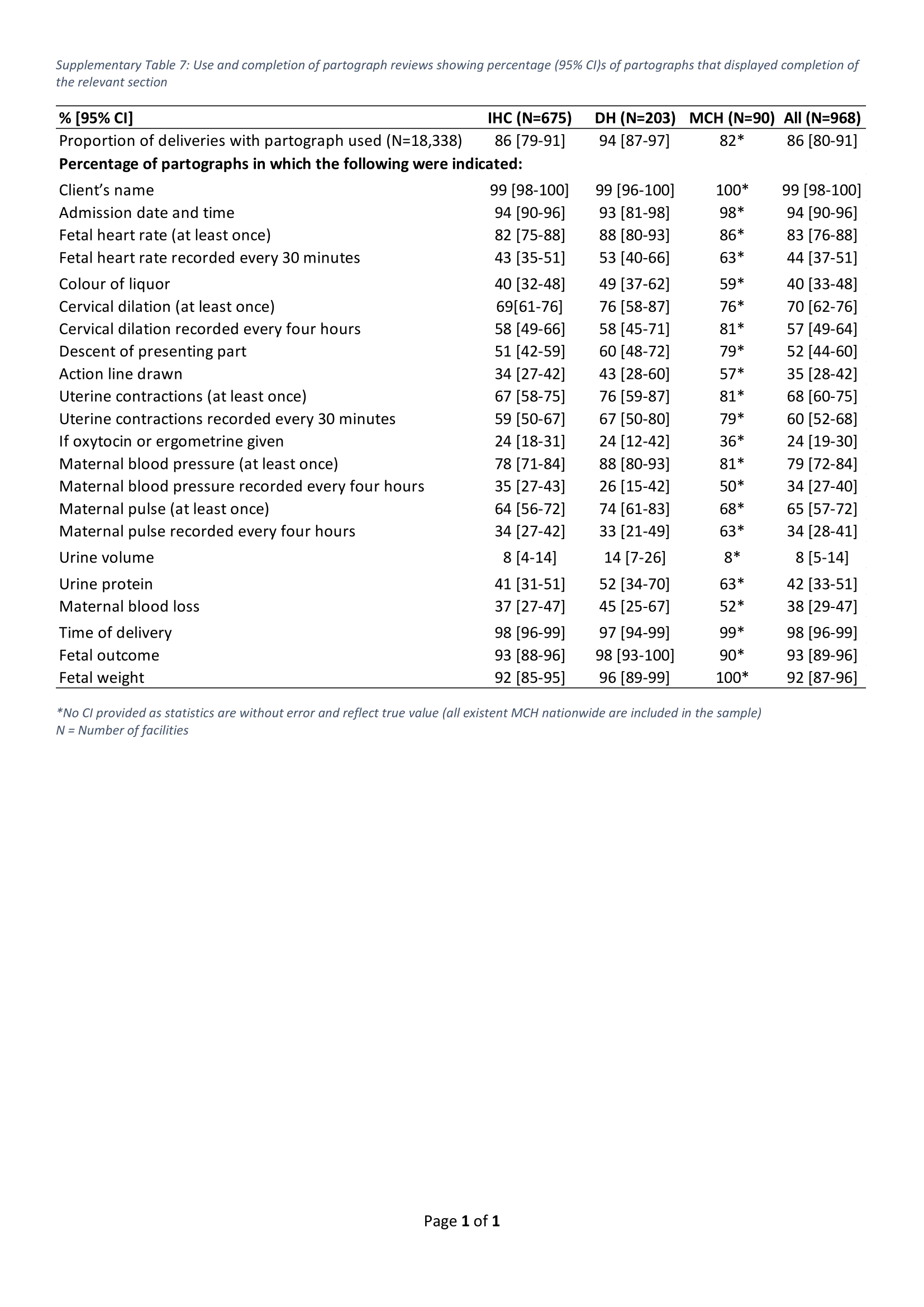

Supplement: S7 Table — (TIFF) [file pgph.0003268.s009.tiff]
